# Study on the mechanism of dual academic research pressure on anxiety among master’s students under an involution context: evidence from a survey of 46 Chinese universities

**DOI:** 10.3389/fpsyg.2025.1667922

**Published:** 2025-11-19

**Authors:** Hang Shang, Tongcan Gao, Lixia Niu

**Affiliations:** School of Business Administration, Liaoning Technical University, Huludao, China

**Keywords:** dual academic research pressure among master’s students, master’s student anxiety, psychological resilience, achievement motivation, dual-pathway research

## Abstract

**Introduction:**

Under an involution context, and drawing on dual-pressure perception and related theories, the mechanism by which dual academic research pressures influence anxiety among Chinese master’s students was examined.

**Methods:**

The Challenge–Hindrance Stress Scale, the Psychological Resilience Scale, and the Anxiety Perception Measurement Scale were employed. Using a multi-wave longitudinal tracking design, surveys were administered to over 2,000 enrolled master’s students from 46 Chinese universities. SPSS PROCESS was used, with Bootstrap resampling set at 5,000 iterations, to test the proposed moderated dual-pathway mediation model.

**Results:**

Challenge-type research pressure was significantly positively correlated with facilitative anxiety (r = 0.52, *p* < 0.01) and significantly negatively correlated with inhibitory anxiety (r = −0.48, *p* < 0.01). Hindrance-type research pressure was significantly negatively correlated with facilitative anxiety (r = −0.38, *p* < 0.01) and significantly positively correlated with inhibitory anxiety (r = 0.56, *p* < 0.01). Achievement motivation mediated the relationships between challenge-type research pressure and both facilitative and inhibitory anxiety. Psychological resilience mediated the relationships between hindrance-type research pressure and both facilitative and inhibitory anxiety. A promotion-focused regulatory focus positively moderated the positive effect of challenge-type research pressure on achievement motivation. A prevention-focused regulatory focus negatively moderated the negative effect of hindrance-type research pressure on psychological resilience. The promotion-focused regulatory focus enhanced the indirect effect of “challenge-type research pressure → achievement motivation → facilitative anxiety.” The prevention-focused regulatory focus negatively affected the indirect effect of “hindrance-type research pressure → psychological resilience → facilitative anxiety.”

**Conclusion:**

Attention to postgraduate students’ research pressures and anxiety necessitates targeted interventions at multiple levels, including universities, faculties, supervisors, and students themselves.

## 1 Introduction

The involution context adopted in this study derives from Kant’s philosophical concept and, combined with a sociological perspective, is defined as an irrational form of internal competition in which inputs continuously increase without commensurate returns (Sun and Tang, 2023). Notably, as a term with pronounced local cultural character, “involution” resonates with widely observed issues of academic pressure in global higher education, such as burnout and psychological stress (Yan and Zhang, 2024). However, the distinctive theoretical value of “involution” lies in transcending a mere enumeration of stressors. Compared with globally prevalent stress frameworks, the involution perspective places greater emphasis on how a structure of irrational, diminishing-return competition itself becomes an overwhelming situational appraisal. Accordingly, this study posits “involution” as a critical macro-level contextual perception that acts as an amplifier, significantly intensifying the mechanism by which dual-track research pressures exacerbate anxiety among graduate students. This research engages with global scholarship on academic stress, offering a unique empirical contribution to understanding the formation mechanisms of stress across diverse cultural contexts.

Within the increasingly competitive landscape of higher education, university students inevitably face an involution crisis, which manifests not only at the undergraduate level but also significantly at the postgraduate stage. With the continual expansion of postgraduate enrolment, excessive research-related pressure and heightened anxiety among master’s students have become increasingly pronounced. Postgraduate education, serving as a primary channel for cultivating high-level talent, constitutes an essential platform for nurturing top innovative individuals and provides pivotal support to the national strategy of building a talent-powered nation (Zhu et al., 2024). As the national strategy for cultivating top-tier innovative talents and university research evaluation systems undergo continuous reform, state requirements for the academic standards and quality of master’s students have correspondingly escalated, intensifying their research pressures. Such high-intensity academic stress frequently precipitates increased irrational competition within student groups. In an environment permeated by societal involution, master’s students’ lives and studies appear increasingly defined by anxiety and frustration (Stallman, 2010). A recent study indicated that approximately one-quarter of postgraduate students in a former “985 Project” university in Beijing experienced varying degrees of psychological stress and anxiety, with primary stressors centred around academic research and research productivity—particularly pressure associated with completing research projects and publishing in professional journals (Song et al., 2019). Under conditions of involution, master’s students encounter excessive competitive pressures, reflected not merely in academic achievements but also in the pursuit of scarce academic resources, opportunities, and honours. Struggling to obtain limited scholarships, publishing opportunities, or access to distinguished supervisors, students frequently lose sight of the substantive purposes of research. They disproportionately devote excessive time and energy to superficial refinement—such as overly concerning themselves with the format, structure, and length of research papers—instead of engaging in substantial innovation or critical exploration of research problems. Within an involuted environment, master’s students often endure significant psychological stress; they must navigate not only personal research pressures but also high expectations from supervisors and family, compounded further by peer competition. Over time, such pressures risk precipitating anxiety and depression, exacerbating psychological distress, obstructing innovation, and potentially harming their physical and mental health as well as academic development. Therefore, examining the mechanisms through which academic research pressures influence anxiety among master’s students in China holds considerable importance for ensuring their holistic well-being and development. Clarifying the negative emotional consequences of such pressures and harnessing their inherent motivational potential have thus emerged as critical concerns warranting scholarly attention.

## 2 Literature review and research hypotheses

### 2.1 Challenge-type pressure, hindrance-type pressure

Originating from dual-pressure perception theory, challenge-type pressure is appraised as a benign demand with potential gains that fosters personal growth and goal attainment, whereas hindrance-type pressure is appraised as a malignant demand that impedes goal attainment and depletes personal resources (Dai et al., 2020). Contexts characterised by high challenge-type pressure may stimulate achievement motivation, while contexts of high hindrance-type pressure often culminate in emotional exhaustion and withdrawal behaviours. Although the dual-pressure model has yielded valuable findings in organisational behaviour—revealing complex effects on employee performance, innovative behaviour, and burnout—its application to higher-education settings, particularly to research pressures specific to master’s students, remains insufficiently explored. Rodell and Judge (2009) focusing on university students, divided research pressures into challenge-type and hindrance-type. Challenge-type pressure emphasises the setting of appropriate research evaluation standards and expectations, thereby driving greater investment in academic work, promoting research outputs, and stimulating research innovation; it typically shows positive associations with individuals’ psychological states, emotions, and research productivity. Hindrance-type pressure accentuates negative influences such as academic stress, tense supervisor–student relations, heavy research workloads, and constrained personal development—factors that may even incubate academic misconduct—thus obstructing students in achieving research goals or desired states.

### 2.2 Facilitative anxiety, inhibitory anxiety

Anxiety, as a complex emotional experience, is not purely negative in function. According to its influence on goal pursuit, anxiety may be differentiated into two functional types. Facilitative anxiety is associated with the desire for potential gains and progress and may elicit adaptive coping; inhibitory anxiety, by contrast, is linked to fear of potential loss and failure and tends to produce avoidance and dysfunction. Anxiety theory originated in existential philosophy (Lin and Zheng, 2014) and subsequently evolved within psychoanalytic, behaviourist, and cognitive traditions. Traditional research has often defined anxiety as a negative experience—tension and unease arising when goals are anticipated as unattainable (Zhang, 2003)—and has tended to distinguish it along trait or state dimensions (Yang, 2000). Despite numerous valuable findings, prior work has frequently treated anxiety as a single, dysfunctional primitive variable; moreover, scholarly attention has largely concentrated on the adverse effects of inhibitory anxiety, with few studies simultaneously examining facilitative and inhibitory anxiety as outcome variables. Master’s students currently encounter complex, research-driven stressors, and their resulting affective experiences are far from a unitary negative anxiety. At present, research remains limited regarding the specific manifestations and cognitive appraisal processes of these two functionally distinct anxieties within the master’s cohort, as well as their unique mechanisms for shaping research engagement. Consequently, our understanding of how to identify and intervene scientifically in the complex anxiety responses of master’s students is incomplete. Accordingly, this study divides anxiety in research activities into facilitative anxiety and inhibitory anxiety. The former denotes task-driven tension, concentration, and moderate arousal with motivational properties that prompt individuals to overcome difficulties, take on new challenges, and work harder; the latter manifests as anxiety and worry in uncertain situations, with inhibitory cognitive, behavioural responses that interfere with functioning, engender frustration, and precipitate avoidance of study or other activities, possibly culminating in withdrawal (Liu, 2005).

### 2.3 Achievement motivation

Achievement motivation refers to a psychological propensity to accept challenging and demanding tasks, together with the desire to excel or outperform others (Atkinson, 1957). Based on achievement motivation theory, when individuals face challenging research tasks and strong achievement motivation is activated, pressure is more likely to be appraised as an opportunity for growth, prompting adaptive coping and thereby alleviating anxiety; conversely, inadequate achievement motivation increases the risk of disorientation under stress and entrapment in anxiety (Wang and Chang, 2023). Although research on achievement motivation has produced many valuable findings—its general predictive power for academic achievement, its positive influence on career development, and its cross-cultural manifestations—evidence remains limited regarding its specific role in high-pressure academic contexts. In particular, within the master’s student population, research on the distinctive forms, antecedents, and mechanisms of achievement motivation under an involutional research environment remains insufficient.

### 2.4 Psychological resilience

Psychological resilience denotes the capacity to cope with stress healthily and to attain personal goals at minimal psychological and physical cost, thereby exhibiting strong self-recovery (Epstein and Krasner, 2013). Grounded in stress-and-coping theory, when individuals face high-intensity research pressures, a high level of resilience increases the likelihood that pressure will be appraised as a controllable challenge, eliciting adaptive coping and preserving mental health; by contrast, insufficient resilience risks rapid depletion of psychological resources and emotional distress. Although numerous studies have shown that resilience enhances self-efficacy, reduces academic burnout, and improves emotion regulation, research remains limited regarding its specific protective mechanisms in high-pressure academic settings—especially among master’s students—concerning the processes through which resilience functions under intense research pressure and its distinctive mechanisms of influence. This gap constrains our understanding of how best to cultivate and intervene in master’s students’ resilience.

### 2.5 Promotion-focused, prevention-focused regulatory focus

According to affective events theory, individuals’ responses to events in organisational settings are closely linked to personality traits; faced with identical external events, different individuals may exhibit divergent emotional and motivational responses, leading to different outcomes. Dispositional characteristics moderate the link between work events and subsequent responses, and regulatory focus is a key motivational system explaining these differences (Ma et al., 2024). Rooted in self-discrepancy theory, regulatory focus explains the relationship between self-regulatory motivational systems and behavioural strategies in goal pursuit (Weiss and Cropanzano, 1996). Traditionally, it has been examined either as a stable trait or as a situationally induced variable to study effects on task performance or decision outcomes (Higgins et al., 1997). Although the literature is rich, prior studies largely treat regulatory focus as a general motivational variable, most often in workplace or consumer settings, with little examination of its specific manifestations in high-pressure academic contexts (Higgins, 1997). Master’s students confront complex academic tasks that jointly involve aspiration and responsibility-avoidance; their motivational tendencies are therefore not reducible to a single trait. Current research remains limited regarding how these two functionally distinct systems manifest within this cohort, constraining understanding of how to stimulate and guide research motivation effectively. Consequently, trait regulatory focus is divided here into promotion-focused and prevention-focused orientations. The former is a gain-approach focus: individuals pursue achievement and growth, emphasize goal attainment and growth motivation, and display openness and proactive behaviour. The latter is a loss-avoidance focus: individuals emphasise responsibilities and obligations, attend to security and duty fulfilment, think cautiously and conservatively, and behave defensively to avoid risk.

### 2.6 Dual academic research pressure and facilitative vs. inhibitory anxiety

Dual-pressure perception theory centres on how individuals appraise and cope with different types of pressure. Cavanaugh et al. (2000) via meta-analysis, divided postgraduate stressors into challenge-type and hindrance-type, which exert distinct effects on innovation and psychological states. According to pressure interaction theory (Monat and Lazarus, 1991), individuals may appraise identical situations differently. In this study, individual factors such as personality traits and self-efficacy, and environmental factors such as institutional research assessment standards, supervisory practices, and the availability of research resources, directly shape master’s students’ stress appraisals. Yao and Ma (2021) found that challenge-type pressure negatively affects university students’ anxiety, whereas hindrance-type pressure positively affects anxiety. Yao et al. (2023) showed that research pressure influences depressive mood via the mediating roles of anxiety and burnout. Pu et al. (2024) revealed the mechanism by which perceived stress affects military cadets’ anxiety and confirmed the mediating roles of cognitive reappraisal and expressive suppression. Li et al. (2024) reported that learning, interpersonal, and uncertainty pressures are all positively correlated with anxiety, with uncertainty exerting the strongest effect. Zhang et al. (2023) showed that challenge-type time pressure significantly fosters breakthrough creativity, whereas hindrance-type time pressure significantly constrains it. Gu et al. (2024) identified occupational stress as a risk factor for anxiety and depression among female managers. Yan and Li (2023) found a significant positive correlation between research pressure and depression. Webster et al. (2010) argued that hindrance stressors evoke negative emotions such as anxiety, frustration, and anger.

Based on the research findings H1: Challenge-type research pressure positively predicts facilitative anxiety; challenge-type research pressure negatively predicts inhibitory anxiety. Hindrance-type research pressure negatively predicts facilitative anxiety; hindrance-type research pressure positively predicts inhibitory anxiety.

### 2.7 Achievement motivation and psychological resilience

According to cognitive appraisal theory, individuals’ evaluations of their environments are central to emotional and behavioural responses. Wang et al. (2014) reported that when challenge-type pressure is appraised as a positive opportunity, intrinsic achievement motivation is activated, thereby exerting a significant positive effect on performance. Liu et al. (2021) showed that challenge-type pressure stimulates young teachers’ achievement motivation. When master’s students appraise research pressure as challenging, such pressure becomes a catalyst for activating achievement motivation; thus, challenge-type research pressure positively predicts achievement motivation. In addition, achievement motivation, as a key internal psychological resource, has been found in some studies to positively influence examination anxiety, whereas other work shows a significant negative association with state anxiety.

Individuals high in achievement motivation tend to set higher future goals (Zhang et al., 2022) and are more likely to transform pressure into goal-directed drive. Hence, the observed positive association likely reflects functional, problem-solving facilitative anxiety. Challenge-type pressure, by activating achievement motivation, sustains an appropriate level of tension and focus; this anxiety is motivationally activating (Liu et al., 2024). Individuals high in achievement motivation tend to be proactive and confident (Su and Dong, 2015); when encountering difficulties, this positive tendency makes them less prone to fear of uncertainty and cognitive inhibition (Chen, 2023). Accordingly, the observed negative association reflects a buffering effect of achievement motivation on dysfunctional inhibitory anxiety. As a protective resource, achievement motivation can effectively prevent challenge-type pressure from transforming into inhibitory anxiety characterised by frustration and avoidance (Song, 2024).

Based on the research findings H2: Achievement motivation mediates the relationship between challenge-type research pressure and facilitative anxiety. Achievement motivation also mediates the relationship between challenge-type research pressure and inhibitory anxiety.

Based on cognitive appraisal theory, hindrance-type research pressure is appraised as a threatening demand that obstructs goal attainment. Prolonged exposure to such pressure continually consumes psychological resources. Psychological resilience, as a positive psychological resource, enables individuals to cope with adversity (Epstein and Krasner, 2013), yet it is not inexhaustible. When hindrance-type pressure is excessive, cognitive and affective resources are heavily occupied in threat management, leading to pronounced depletion of resilience as an internal resource (Wang et al., 2010).

Moreover, resilience is a pivotal hub linking pressure and emotional experience. Prior studies indicate that resilience exerts distinct effects on facilitative and inhibitory anxiety. First, resilience positively predicts emotional stability, enabling individuals to maintain positive affective experiences under pressure (Ma et al., 2010). This capacity allows highly resilient individuals, when facing hindrance-type stressors, to problematise rather than catastrophise—cognitively construing them as issues to be solved—which in turn elicits moderate, task-oriented facilitative anxiety (Florez et al., 2020). Second, the core function of resilience is buffering (Xu et al., 2023). Highly resilient individuals cope better with adversity, effectively mitigating depression and anxiety induced by negative life events. Thus, resilience can buffer the sense of threat produced by hindrance-type pressure and significantly reduce inhibitory anxiety characterised by avoidance (Chen, 2024).

Based on the research findings H3: Psychological resilience mediates the relationship between hindrance-type research pressure and facilitative anxiety. Psychological resilience also mediates the relationship between hindrance-type research pressure and inhibitory anxiety.

### 2.8 Trait regulatory focus, dual academic research pressure, and facilitative vs. inhibitory anxiety

In line with affective events theory, individuals exposed to identical events in organisational activities exhibit divergent emotional and cognitive responses owing to personality differences. Substantial evidence shows that dispositional traits significantly moderate the pathways by which the external environment shapes cognition and behaviour (Lanaj et al., 2012). It is posited here that trait regulatory focus moderates the links between dual pressures and the mediators. First, the moderating role of promotion-focused regulatory focus in the relationship between challenge-type research pressure and achievement motivation (Li, 2016): when master’s students are high in promotion focus and encounter challenge-type research pressure, situational cues align closely with their trait motivation, more strongly activating intrinsic achievement motivation (Yu, 2023). Second, as a situational factor that depletes psychological resilience, hindrance-type research pressure aligns with the prevention-focused motivational system of avoidance (Zhang, 2022). When master’s students high in prevention-focused regulatory focus face hindrance-type pressure, situational cues likewise match their trait motivation, heightening threat vigilance; additional psychological resources must be expended, thereby accelerating depletion of resilience (Song et al., 2021).

Based on the research findings H4: Promotion-focused regulatory focus positively moderates the effect of challenge-type research pressure on achievement motivation.

Based on the research findings H5: Prevention-focused regulatory focus negatively moderates the effect of hindrance-type research pressure on psychological resilience.

A strong promotion focus significantly enhances the efficiency with which challenge-type pressure is transformed into achievement motivation. Heightened achievement motivation will in turn influence subsequent anxiety responses. High achievement motivation is cognitively and affectively aligned with facilitative anxiety; when promotion focus enables individuals to derive stronger achievement motivation from challenge, this motivation is efficiently transformed into positive, goal-directed facilitative anxiety. Accordingly, the positive indirect effect along “challenge-type research pressure → achievement motivation → facilitative anxiety” will be significantly strengthened. When promotion focus substantially amplifies achievement motivation, this drive more effectively attenuates inhibitory anxiety. Hence, the negative indirect effect along “challenge-type research pressure → achievement motivation → inhibitory anxiety” will become stronger.

Based on the research findings H6: Promotion-focused regulatory focus positively moderates the indirect effect “challenge-type research pressure → achievement motivation → facilitative anxiety,” and negatively moderates the indirect effect “challenge-type research pressure → achievement motivation → inhibitory anxiety.”

Next, the moderated-mediation effects of prevention focus are derived for the hindrance-type pressure pathway. A strong prevention focus markedly accelerates the depletion of resilience under hindrance-type pressure; this resource loss exerts direct downstream impacts on anxiety. When high prevention focus precipitates rapid collapse of resilience under hindrance-type pressure, the inhibitory effect of hindrance-type pressure on facilitative anxiety is magnified. Thus, the negative indirect effect along “hindrance-type research pressure → psychological resilience → facilitative anxiety” becomes stronger. Under high prevention focus, rapid depletion of resilience leaves individuals fully exposed to the threat posed by hindrance-type pressure, with failure-avoidant cognition fully activated; at this juncture, the perceived threat markedly amplifies inhibitory anxiety. Therefore, the positive indirect effect along “hindrance-type research pressure → psychological resilience → inhibitory anxiety” is significantly strengthened.

Based on the research findings H7: Prevention-focused regulatory focus negatively moderates the indirect effect “hindrance-type research pressure → psychological resilience → facilitative anxiety,” and positively moderates the indirect effect “hindrance-type research pressure → psychological resilience → inhibitory anxiety.”

**Figure 1:**
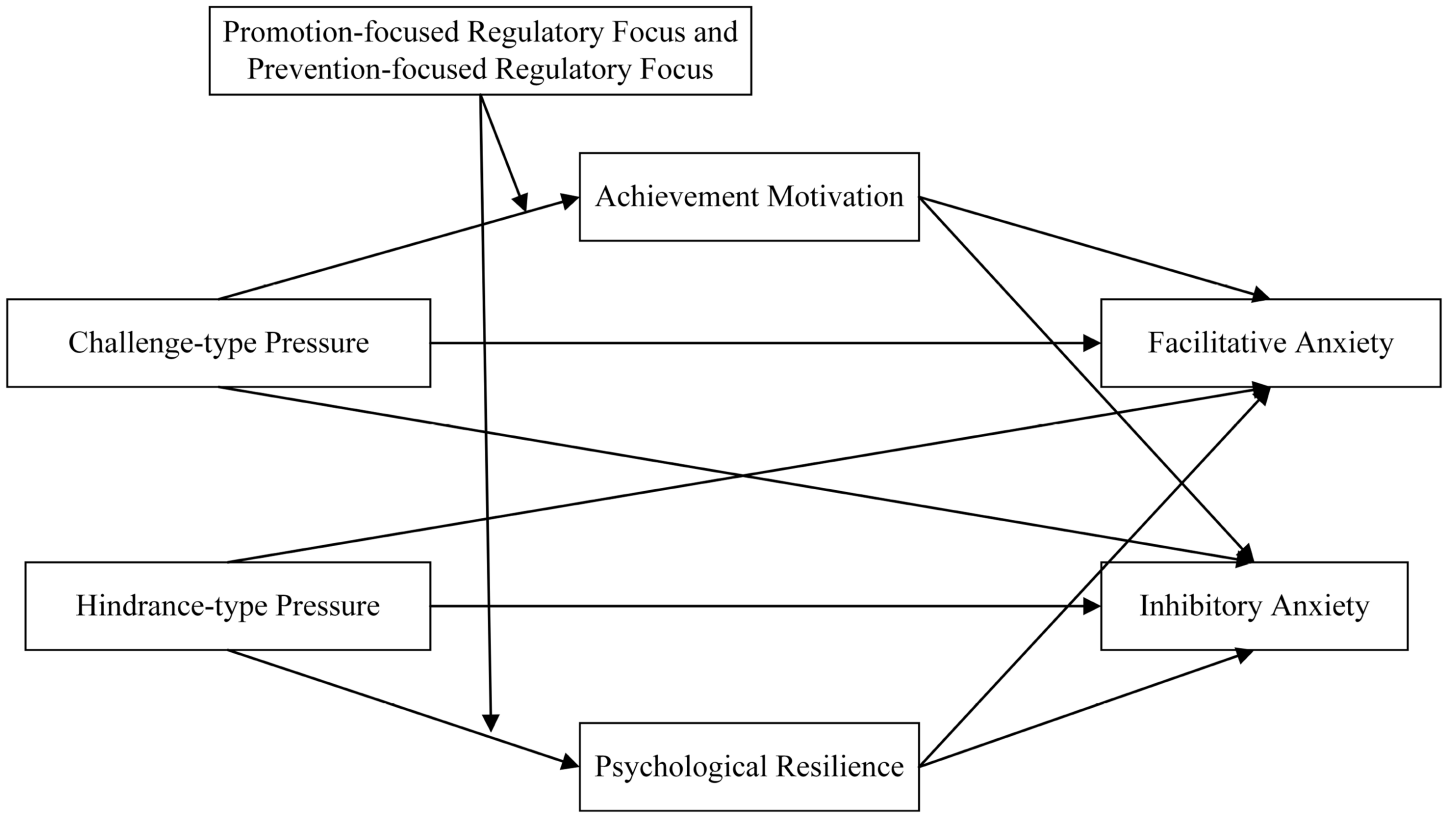
Dual-pathway model.

Based on the foregoing hypotheses, and as shown in Figure 1, a moderated mediation model is constructed to examine, within the current involutional academic environment, the mechanisms by which dual academic research pressures influence dual-dimensional anxiety among Chinese master’s students. Challenge-type and hindrance-type research pressures are treated as the core independent variables, with facilitative and inhibitory anxiety as the ultimate dependent variables. The model’s core mechanism comprises two parallel mediation paths: (i) achievement motivation mediating between challenge-type research pressure and anxiety; and (ii) psychological resilience mediating between hindrance-type research pressure and anxiety. To delineate boundary conditions, trait regulatory focus is introduced as a moderator to test its role on the first-stage transmission of pressure and to further examine its moderating influence across the entire mediation chain, thereby forming a moderated mediation effect.

## 3 Research participants and methods

### 3.1 Research participants and procedure

The participants were full-time master’s students enrolled at 46 universities across seven provinces and municipalities in China (Liaoning, Fujian, Henan, Jiangsu, Guangdong, Beijing, and Tianjin). To ensure representativeness, a stratified random sampling strategy was adopted. First, stratification was conducted by institutional tier and geographical region; universities were then randomly sampled within each stratum, and participants at selected institutions were recruited into a multi-wave longitudinal tracking study.

A three-wave data-collection design was employed to reduce common-method bias and to test time-lag effects among variables. Data collection was completed by the research team at Liaoning Technical University from 1 May 2024 to 1 December 2024. In collaboration with research partners at each university, and after securing permissions from relevant schools and faculties, an electronic informed-consent form was provided to all potential participants via a Wenjuanxing online questionnaire link. Collaborators delivered standardised instructions to all potential participants, detailing the study aims and emphasising confidentiality and voluntariness. To match participants’ data across T1, T2, and T3, informed consent was obtained before questionnaire distribution, and participants were informed that the system would require either an e-mail address used solely for tracking purposes or the creation of a personal anonymous code. It was assured that all matching information would be stored separately in encrypted form, would never be linked to questionnaire responses, and that no personally identifiable information would ever be disclosed. Participants could withdraw from the survey at any time, including during T2 or T3, without providing reasons, and their responses would have no impact on their studies. All data would be kept strictly confidential, used solely for academic analysis, and reported only in aggregate. No foreseeable major risks were involved; the only potential inconvenience was the time required to complete the multi-wave questionnaires. No minors or vulnerable groups were involved. All participants confirmed, by clicking ‘Agree’ on the T1 questionnaire landing page, that they had read and understood all information, voluntarily consented to participate in the study, and consented to the research team’s use of their anonymised questionnaire data. During the study period, 2,218 questionnaires were distributed and returned at T1. Attrition occurred at T2 and T3 due to loss of contact or voluntary withdrawal. After excluding incomplete data, patterned responding, and responses completed in unreasonably short times, 2,000 matched questionnaires completed across all three waves were retained, yielding a valid tracking rate of 90.17%. The final sample comprised 856 males and 1,144 females; 826 academic master’s students and 1,174 professional master’s students; 334 from former ‘985 Project’ universities, 515 from former ‘211 Project’ universities, and 1,151 from other general universities.

### 3.2 Research instruments

The core variables of this study include measurements of challenge-type and hindrance-type pressures, psychological resilience, achievement motivation, and anxiety perceptions. All scales utilised have been sourced from authoritative international and domestic journals, and their reliability has been verified. When translating the original scales, careful adjustments were made in wording and sentence order without altering the original intent, to better align with local linguistic habits and expressions.

#### 3.2.1 Measurement of challenge-type and hindrance-type pressures

The measurements of challenge-type and hindrance-type pressures were adapted from the “Dual Work Stress Measurement Tool” developed by Cavanaugh et al. (2000). In revising and applying the scale within this study, the items were specifically contextualised to the research tasks of master’s students, including literature review, project progression, thesis writing, and publication of results, such as “efforts made to achieve research standards can promote personal growth and well-being,” or “task demands offer challenges in pursuing personal goals and achievements.” This subscale consists of three items, with an internal consistency coefficient of 0.833 and an average variance extracted AVE of 0.718. Conversely, hindrance-type research pressure refers to obstructive demands arising in research practices due to inadequate resources, task conflicts, or unclear procedures. Representative items include “task assignments consume excessive time, interfering with personal goals and developmental processes,” and “meeting task requirements may hinder personal growth and happiness.” Similarly, this subscale comprises three items, with an internal consistency coefficient of 0.886 and an AVE of 0.723. Although the original scale was intended for organisational work settings, all items were localised and contextually adapted for the “research tasks” setting in the current study to ensure measurement validity and specificity for master’s students’ academic research pressures.

#### 3.2.2 Measurement of psychological resilience and achievement motivation

Psychological resilience and achievement motivation, the two core psychological variables measured in this study, are considered critical mediating factors between dual research pressures and anxiety. The Psychological Resilience Scale (Connor–Davidson Resilience Scale, CD-RISC), originally developed by Connor and Davidson (2003) and adapted into Chinese by Yu and Zhang (2007) includes dimensions such as toughness, self-efficacy, and positive coping strategies. It evaluates individuals’ psychological resilience when confronted with stress and uncertainty, showing good measurement validity with an internal consistency coefficient of 0.927 and an AVE of 0.589. Achievement motivation was measured using the Achievement Motivation Scale developed by Yeh et al. (1992) comprising five items assessed via a five-point Likert scale (1 = strongly disagree, 5 = strongly agree). Higher scores indicate stronger internal drive for achieving goals and accomplishments. This scale exhibited an internal consistency coefficient of 0.915 and an AVE of 0.684.

#### 3.2.3 Measurement of anxiety perceptions

Facilitative anxiety was measured using the Anxiety Perception Scale developed by Parker and DeCotiis (1983). The facilitative anxiety subscale showed an internal consistency of 0.834 and an AVE coefficient of 0.558. Inhibitory anxiety was measured using the state-anxiety dimension of the anxiety scale proposed by Carleton et al. (2007). The inhibitory anxiety subscale showed an internal consistency of 0.868 and an AVE coefficient of 0.624. All scales adopted a five-point Likert format. In addition to the primary research variables, the study specifically considered respondents’ gender, only-child status, academic year, disciplinary category, and stage of master’s programme. These factors were incorporated into the research model as control variables to ensure the precision and reliability of the statistical conclusions.

### 3.3 Data processing

A range of statistical analyses was used to test the research hypotheses. Confirmatory factor analysis was conducted with SPSS 26.0 to evaluate the structural validity of the scales employed in this study. Reliability analyses were then performed for each scale, with Cronbach’s *α* coefficients for all variables exceeding the 0.7 criterion.

## 4 Results and analysis

### 4.1 Common-method bias test

To assess the extent of common-method bias, Harman’s single-factor test was employed. SPSS was used for the analysis. The results showed that more than one factor had eigenvalues greater than 1, and the first factor accounted for 44.911% of the variance, which is below the 50% threshold. It may thus be inferred that no single factor explained the majority of the variance and common-method bias was not serious. Multicollinearity diagnostics were performed on the variables, with the degree of collinearity among variables assessed by calculating the variance inflation factor. All variables exhibited VIF values below 3.0, well below the commonly used threshold of 10, indicating no significant multicollinearity issues exist among the variables.

### 4.2 Discriminant validity analysis

Confirmatory factor analysis indicated that the absolute, incremental, and parsimonious fit indices all met the recommended thresholds, demonstrating good model fit: χ^2^/df = 1.852 (< 3.0), CFI = 0.962 (> 0.95), TLI = 0.951 (> 0.95), RMSEA = 0.048 (< 0.05). The results are shown in Table 1.

**Table 1:** Results of confirmatory factor analysis.

Factor model	χ^2^/df	χ^2^	df	TLI	CFI	RMSEA
Eight-factor model	1.852	512.336	277	0.951	0.962	0.048
Five-factor model	2.451	812.551	332	0.892	0.908	0.078
Four-factor model	3.126	1124.782	360	0.832	0.851	0.095
Three-factor model	4.235	1589.441	375	0.761	0.783	0.118
Two-factor model	5.892	2245.672	381	0.682	0.712	0.142
Single-factor model	7.341	2856.334	389	0.598	0.634	0.168

### 4.3 Descriptive statistics and correlation analysis

To examine the correlations among challenge-type research pressure and hindrance-type research pressure, facilitative anxiety and inhibitory anxiety, achievement motivation, psychological resilience, promotion-focused regulatory focus, and prevention-focused regulatory focus, Pearson correlation coefficients were computed to quantify the strength of associations. The analysis indicated that the correlation coefficients among the seven items were significant. Means, standard deviations, and correlation coefficients for each variable are presented in Table 2.

**Table 2:** Correlation analysis.

Variable	M	SD	1	2	3	4	5	6	7	8
Challenge-type pressure	4.25	0.89	1							
Hindrance-type pressure	3.82	0.94	−0.43**	1						
Facilitative anxiety	4.12	0.87	0.52**	−0.38**	1					
Inhibitory anxiety	3.68	0.91	−0.48**	0.56**	−0.45**	1				
Achievement motivation	4.35	0.82	0.65**	−0.41**	0.58**	−0.52**	1			
Psychological Resilience	4.18	0.85	0.39**	−0.55**	0.42**	−0.59**	0.44**	1		
Promotion-focused focus	4.28	0.83	0.51**	−0.35**	0.49**	−0.43**	0.61**	0.38**	1	
Prevention-focused regulatory focus	3.75	0.88	−0.36**	0.58**	−0.34**	0.54**	−0.39**	−0.52**	−0.41**	1

*p < 0.05, **p < 0.01.

### 4.4 Testing direct and mediating effects

#### 4.4.1 Direct effect tests

Table 2 indicates that challenging research pressure is significantly positively correlated with facilitative anxiety (r = 0.52, *p* < 0.01) and significantly negatively correlated with inhibitory anxiety (r = −0.48, *p* < 0.01). Impeding research pressure is significantly negatively correlated with facilitative anxiety (r = −0.38, *p* < 0.01) and significantly positively correlated with inhibitory anxiety (r = 0.56, *p* < 0.01), thereby validating the first research hypothesis.

#### 4.4.2 Testing for mediating effects

This study employed the PROCESS macro with 5,000 bootstrap resamples to test the mediating effects of Achievement Motivation and Psychological Resilience between dual-dimensional research stress and dual-dimensional anxiety. The results are shown in Table 3. The test results confirmed the significant mediating role of Achievement Motivation in the influence of Challenge-type Pressure on both types of anxiety. For Facilitative Anxiety, the indirect effect of Challenge-type Pressure on Facilitative Anxiety through Achievement Motivation was significant, with an effect size of 0.207** and a 95% Bootstrap confidence interval of [0.148, 0.266]. Since the confidence interval does not contain zero, this indicates that Achievement Motivation, as a key hub in the motivational activation pathway, transforms challenging pressure into a positive driving force. For Inhibitory Anxiety, the indirect effect of Challenge-type Pressure on Inhibitory Anxiety through Achievement Motivation was equally significant, with an effect size of −0.230** and a 95% Bootstrap confidence interval of [−0.299,-0.161]. This interval excludes zero, indicating that high Achievement Motivation, as a protective psychological resource, effectively buffers and attenuates the impact of challenging stress on Inhibitory Anxiety. In summary, the second hypothesis is fully supported.

**Table 3:** Mediation effect analysis.

Path	Indirect effect	Boot SE	Boot 95% CI
Challenge-type pressure → achievement motivation → facilitative anxiety	0.207**	0.031	[0.148, 0.266]
Challenge-type pressure → achievement motivation → inhibitory anxiety	−0.230**	0.036	[−0.299, −0.161]
Hindrance-type pressure → psychological resilience → facilitative anxiety	−0.246**	0.034	[−0.312, −0.180]
Hindrance-type pressure → psychological resilience → inhibitory anxiety	0.271**	0.038	[0.197, 0.345]

**p < 0.01.

The test results confirmed the significant mediating role of Psychological Resilience in the effects of Hindrance-type Pressure on both types of anxiety. For Facilitative Anxiety, the indirect effect of Hindrance-type Pressure on Facilitative Anxiety through Psychological Resilience was significant, with an effect size of −0.246** and a 95% Bootstrap confidence interval of [−0.312, −0.180]. This confidence interval excludes zero, and the negative effect indicates that Hindrance-type Pressure significantly depletes Psychological Resilience—a critical resource—thereby weakening individuals’ capacity to generate positive Facilitative Anxiety. For Inhibitory Anxiety, the indirect effect of Hindrance-type Pressure on Inhibitory Anxiety through Psychological Resilience was equally significant, with an effect size of 0.271** and a 95% Bootstrap confidence interval of [0.197, 0.345]. This interval does not include zero, indicating that the failure of Psychological Resilience as a buffering mechanism allows the threat perception from Hindrance-type Pressure to directly translate into Inhibitory Anxiety, thereby intensifying the individual’s psychological distress. In summary, the third hypothesis is fully supported.

#### 4.4.3 Adjustment of intermediate inspection

This study employed hierarchical multiple regression analysis to examine the moderating effect of trait-focused regulation. All data analyses were conducted after controlling for demographic variables, including gender, grade level, master’s program type, and university category. To avoid multicollinearity issues, all independent variables and moderator variables underwent centering before constructing interaction terms. The results are shown in Table 4. After controlling for variables, Challenge type Pressure, and Promotion-focused Regulatory Focus in Model 1, the model was significant (*F* = 38.247, *p* < 0.01), with an adjusted R^2^ of 0.428. The results indicate that both Challenge type Pressure (*β* = 0.418, *p* < 0.01) and Promotion focused Regulatory Focus (β = 0.326, *p* < 0.01) significantly and positively predict Achievement Motivation. The interaction term also significantly predicted Achievement Motivation (β = 0.268, *p* < 0.01). The adjusted R^2^ of Model 2 increased to 0.512, with the change in *F* value reaching statistical significance (*p* < 0.01). Thus, it was concluded that Promotion focused Regulatory Focus exerted a significant positive moderating effect between Challenge-type Pressure and Achievement Motivation. Consequently, the fourth hypothesis was supported.

**Table 4:** Results of moderation analysis.

Dependent variables	Achievement motivation		Psychological resilience	
	Model 1	Model 2	Model 1	Model 2
Gender	−0.042	−0.045	0.028	0.025
Academic year	0.035	0.032	−0.021	−0.018
Type of Master’s program	−0.038	−0.036	0.015	0.012
Type of institution	0.026	0.024	−0.031	−0.029
Challenge-type pressure	0.418**	0.285**		
Promotion-focused regulatory focus	0.326**	0.294**		
Challenge-type pressure × promotion-focused regulatory focus		0.268**		
Hindrance-type pressure			−0.452**	−0.316**
Prevention-focused regulatory focus			−0.385**	−0.348**
Hindrance-type pressure × prevention-focused regulatory focus				−0.294**
Adjusted R^2^	0.428	0.512	0.385	0.468
F	38.247**	45.632**	32.156**	40.328**

**p < 0.01.

Hindrance-type Pressure (β = −0.452, *p* < 0.01) and Prevention-focused Regulatory Focus (β = −0.385, *p* < 0.01) both significantly negatively predicted Psychological Resilience. Results indicated that the interaction term significantly predicted Psychological Resilience (β = −0.294, *p* < 0.01). The adjusted R^2^ of Model 2 increased to 0.468, with the change in F value reaching statistical significance (*p* < 0.01). Thus, Prevention-focused Regulatory Focus exerted a significant negative moderating effect between Hindrance-type Pressure and Psychological Resilience. Consequently, the fifth hypothesis was supported.

**Figure 2:**
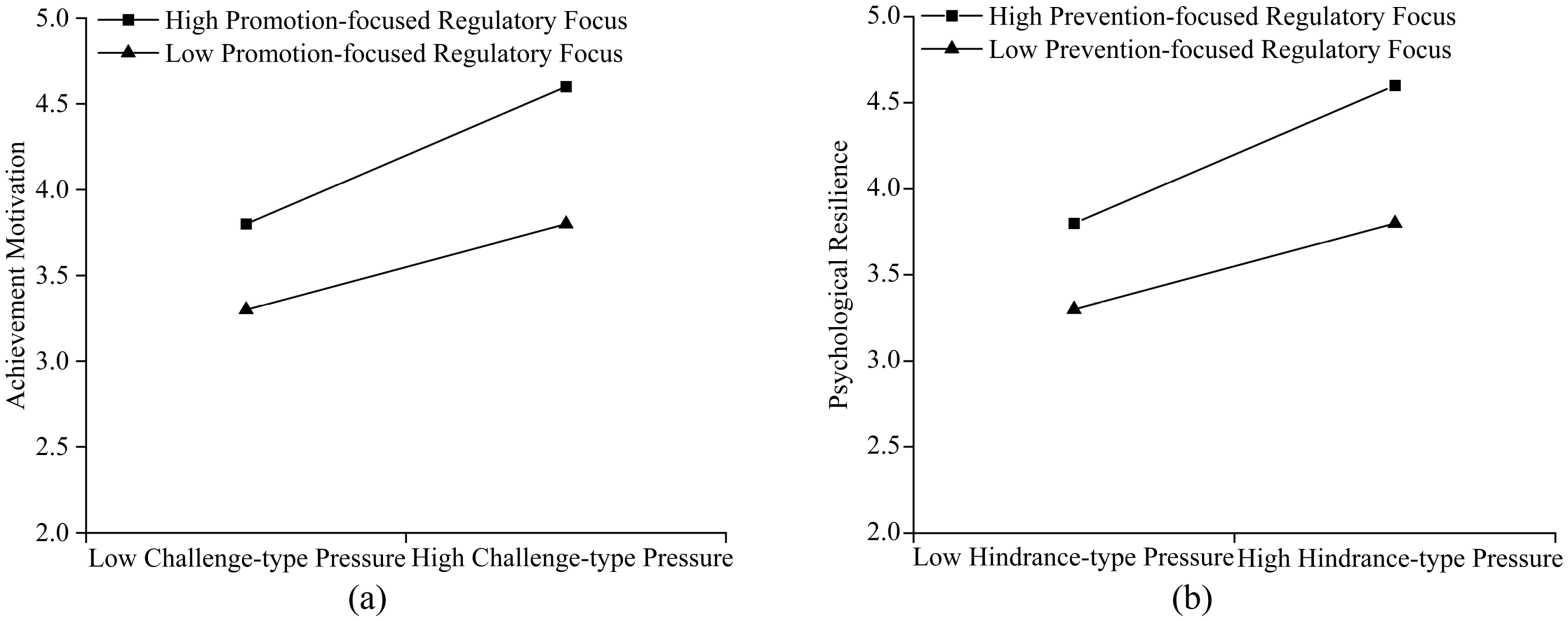
Effects of trait regulatory focus. **(a)** The moderating effect of promotion focused regulatory focus on the relationship between challenge-type pressure and achievement motivation. **(b)** The moderating effect of prevention focused regulatory focus on the relationship between hindrance-type pressure and psychological resilience.

Figure 2 visually illustrates the moderating effects of trait regulatory focus on the first two pathways. In Figure 2a, a Promotion-focused Regulatory Focus positively moderates the positive effect of Challenge type Pressure on Achievement Motivation. Individuals with high Promotion-focused Regulatory Focus are better at viewing Challenge-type Pressure as an opportunity, thereby more effectively stimulating their own Achievement Motivation. In Figure 2b, the Prevention-focused Regulatory Focus negatively moderates the impact of Hindrance-type Pressure on Psychological Resilience. Individuals with a high Prevention-focused Regulatory Focus, when confronted with Hindrance-type Pressure, may be more inclined to focus on potential risks and failures. This leads to an excessive depletion of their psychological resources, resulting in lower Psychological Resilience.

To examine the significance of the moderating effect, this study conducted Simple Slope tests and slope difference tests. As shown in Table 5, the influence of dual pressures on Achievement Motivation and Psychological Resilience exhibited significant differences across varying levels of trait-based regulatory focus. When individuals exhibited a low Promotion-focused Regulatory Focus, the positive predictive effect of Challenge type Pressure on Achievement Motivation reached statistical significance (β = 0.186, *p* < 0.001). Conversely, at high Promotion-focused Regulatory Focus levels, the positive motivational effect of Challenge-type Pressure on Achievement Motivation became more pronounced and intense (β = 0.524, *p* < 0.001). At low Prevention-focused Regulatory Focus levels, the negative impact of Hindrance-type Pressure on Psychological Resilience was significant (β = −0.203, *p* < 0.001). Conversely, when individuals exhibited a high Prevention-focused Regulatory Focus, the negative impact of Hindrance-type Pressure on Psychological Resilience intensified, with a significant increase in the absolute value of the negative coefficient (β = −0.587, *p* < 0.001).

**Table 5:** Results of simple slope tests and slope difference tests.

Path	Regulatory focus level (±1 SD)	Effect size	SE
Challenge-type pressure → achievement motivation	Low promotion-focused regulatory focus (-1SD)	0.186**	0.038
	High promotion-focused regulatory focus (+1SD)	0.524**	0.041
	Difference	0.338**	0.052
Hindrance-type pressure → psychological resilience	Low prevention-focused regulatory focus (-1SD)	−0.203**	0.036
	High prevention-focused regulatory focus (+1SD)	−0.587**	0.039
	Difference	−0.384**	0.048

This study further employed the Bootstrap method to examine the moderating effect of trait-focused attention on the mediating pathway within the model, specifically testing the moderated mediating effect. The results are presented in Table 6. The first part of Research Hypothesis 6 indicates that a Promotion-focused Regulatory Focus exerts a significant positive moderating effect on this pathway. At low Promotion focused Regulatory Focus, the indirect effect is significant with an effect size = 0.128, 95%CI [0.082, 0.176]. At high Promotion focused Regulatory Focus, this indirect effect significantly increased (effect size = 0.286, 95%CI [0.226, 0.348]). The difference between high and low focus groups was 0.158, with a 95% confidence interval of [0.089, 0.227]. This interval does not include zero, indicating that the positive moderating effect of Promotion-focused Regulatory Focus is significant. The second half of Research Hypothesis 6 also demonstrated a significant moderating effect of Promotion-focused Regulatory Focus on this pathway. At low Promotion focused Regulatory Focus, the negative indirect effect was significant, with an effect size of −0.142 and a 95%CI of [−0.194, −0.091]. At high levels of Promotion-focused Regulatory Focus, this negative indirect effect intensified, with an effect size of −0.318 and a 95%CI of [−0.385, −0.251]. The difference between the low and high focus groups was −0.176, with a 95% confidence interval of [−0.251, −0.101]. This interval does not include zero, indicating that as the Promotion-focused Regulatory Focus intensifies, the negative impact of this indirect effect becomes stronger, confirming a negative regulatory effect.

**Table 6:** Bootstrap test results for moderated mediation effects.

Path	Moderator level	Effect size	Boost SE	Boot 95% CI
Challenge-type pressure → achievement motivation → facilitative anxiety	Low promotion-focused regulatory focus (-1SD)	0.128	0.024	[0.082, 0.176]
	High promotion-focused regulatory focus (+1SD)	0.286	0.031	[0.226, 0.348]
	Difference	0.158**	0.035	[0.089, 0.227]
Challenge-type pressure → achievement motivation → inhibitory anxiety	Low promotion-focused regulatory focus (-1SD)	−0.142	0.026	[−0.194, −0.091]
	High promotion-focused regulatory focus (+1SD)	−0.318	0.034	[−0.385, −0.251]
	Difference	−0.176**	0.038	[−0.251, −0.101]
Hindrance-type pressure → psychological resilience → facilitative anxiety	Low prevention-focused regulatory focus (-1SD)	−0.168	0.028	[−0.223, −0.113]
	High prevention-focused regulatory focus (+1SD)	−0.325	0.033	[−0.390, −0.260]
	Difference	−0.157**	0.036	[−0.228, −0.086]
Hindrance-type pressure → psychological resilience → inhibitory anxiety	Low prevention-focused regulatory focus (-1SD)	0.185	0.030	[0.126, 0.244]
	High prevention-focused regulatory focus (+1SD)	0.358	0.035	[0.289, 0.427]
	Difference	0.173**	0.039	[0.096, 0.250]

In the first half of Hypothesis 7, Prevention-focused Regulatory Focus exerted a significant negative moderating effect on this pathway. At low levels of Prevention-focused Regulatory Focus, the indirect effect was negative, with an effect size of −0.168 and a 95%CI of [−0.223,-0.113]. At high Prevention-focused Regulatory Focus, this negative indirect effect significantly intensified, with an effect size of −0.325 and a 95%CI of [−0.390, −0.260]. The difference between the high and low focus groups was −0.157, with a 95% confidence interval of [−0.228, −0.086]. This interval does not include zero, indicating that the negative moderating effect of Prevention-focused Regulatory Focus is significant. The second half of Research Hypothesis 7 also demonstrated a significant moderating effect of Prevention-focused Regulatory Focus on this pathway. At low levels of Prevention-focused Regulatory Focus, this positive indirect effect was significant, with an effect size of 0.185 and a 95%CI [0.126, 0.244]. At high Prevention focused Regulatory Focus, this positive indirect effect significantly increased, with an effect size of 0.358 and 95%CI [0.289, 0.427]. The difference between the high and low focus groups was 0.173, with a 95% confidence interval of [0.096, 0.250]. This interval does not include zero, indicating that the positive moderating effect of Prevention-focused Regulatory Focus is significant. In summary, trait-based regulatory focus consistently served as a boundary condition, significantly moderating the indirect effects of dual research pressure on Facilitative Anxiety and Inhibitory Anxiety through different mediating variables. Both Hypothesis 6 and Hypothesis 7 were supported.

## 5 Main conclusions and recommendations

### 5.1 Main conclusions

The core theoretical contribution of this study lies in providing empirical validation at the micro-psychological mechanism level for “involution,” a social context with distinct local cultural characteristics. Unlike the global academic stress framework and traditional dual-stress models, which primarily focus on individuals’ cognitive evaluations of discrete stressors, the concept of “involution” introduced in this study emphasizes a diffuse, irrational perception of macro-level social competition. While traditional models effectively distinguish between “benign” and “malignant” stress, the findings of this study’s dual-path mediation model confirm that the aforementioned macro-level competitive perception acts as a critical contextual variable. It profoundly reshapes the transmission mechanisms of these micro-stress pathways, thereby influencing anxiety generation among master’s degree students.

#### 5.1.1 Dual academic research pressure significantly affects anxiety among master’s students

Dual academic research pressure exerts a pronounced and differentiated impact on master’s students’ anxiety, revealing a clear double-edged sword effect. Challenge-type research pressure significantly and positively predicted facilitative anxiety, whilst significantly and negatively predicting inhibitory anxiety. Hindrance-type research pressure significantly and negatively predicted facilitative anxiety, whilst significantly and positively predicting inhibitory anxiety.

#### 5.1.2 Achievement motivation and psychological resilience play important roles in regulating research pressure among master’s students

This study confirmed that achievement motivation and psychological resilience function as parallel mediators in the effects of dual academic research pressure on anxiety. Achievement motivation is the key pathway for transforming challenge. Challenge-type research pressure markedly stimulated achievement motivation, thereby increasing facilitative anxiety and reducing inhibitory anxiety. Psychological resilience is the key pathway for countering hindrance. Hindrance-type research pressure significantly depleted psychological resilience, thereby weakening facilitative anxiety and exacerbating inhibitory anxiety.

#### 5.1.3 Trait regulatory focus effectively moderates master’s students’ anxiety

Trait regulatory focus moderated the mediating role of achievement motivation. When confronting challenge-type research pressure, individuals’ regulatory focus determined the extent to which achievement motivation was drawn from or drained by pressure, thereby influencing anxiety. Under a promotion-focused orientation, master’s students tended to construe challenge-type pressure as an opportunity for achievement, positively interpreting pressure as a source of drive and, through proactively overcoming difficulties, substantially enhancing achievement motivation. The focus and engagement engendered by high achievement motivation facilitated positive academic feedback and self-efficacy, thereby buffering the transmission of pressure to inhibitory anxiety and potentially converting it into facilitative anxiety. Conversely, when a prevention-focused orientation predominated, students confronting the same challenge-type pressure were more likely to focus on the consequences of failure and error avoidance; motivation thus derived more from fear, an avoidance orientation that impeded the extraction of intrinsic drive from challenge and could inhibit the transformation from challenge-type pressure to achievement motivation.

Trait regulatory focus also moderated the mediating role of psychological resilience. When facing hindrance-type research pressure, differing regulatory foci determined the extent to which resilience was depleted or maintained, thereby influencing anxiety. Under a prevention-focused orientation, master’s students were more inclined to appraise hindrance-type pressure as a direct threat; this cognitive pattern significantly and negatively moderated the consumption of psychological resources by hindrance-type pressure, leading to a sharp decline in resilience. Defensive failure resulting from low resilience impeded effective buffering, thereby markedly strengthening the effect of hindrance-type pressure on inhibitory anxiety. By contrast, when a promotion-focused orientation predominated, students—though also discouraged—were more likely to construe hindrance as an obstacle to be overcome en route to ultimate goals; this approach orientation enabled a more objective appraisal of hindrance and mobilisation of resources. This study confirmed that promotion-focused regulatory focus positively moderated the indirect effect “challenge-type research pressure → achievement motivation → facilitative anxiety,” and negatively moderated the indirect effect “challenge-type research pressure → achievement motivation → inhibitory anxiety.” Prevention-focused regulatory focus negatively moderated the indirect effect “hindrance-type research pressure → psychological resilience → facilitative anxiety,” and positively moderated the indirect effect “hindrance-type research pressure → psychological resilience → inhibitory anxiety.”

### 5.2 Recommendations

Firstly, universities should establish comprehensive postgraduate mental health service systems by setting up professional psychological counselling and academic advising centres, equipped with psychological counsellors and academic development advisor teams. Standardised service protocols and dynamic management systems should be established. Dynamic tracking of postgraduate mental health should be carried out by creating individual psychological health files. For high-risk groups, a three-level “screening–early warning–intervention” response mechanism should be developed, alongside satisfaction surveys and outcome evaluations, thus forming a closed-loop management system incorporating service provision, quality monitoring, and performance improvement to ensure effective and timely mental health support.

Secondly, academic faculties should build a three-dimensional collaborative system covering management, cultivation, and motivation. Regular activities should facilitate dialogues between supervisors and students, optimising master’s student registration processes and establishing dual-track scholarship evaluation standards. A two-channel feedback mechanism comprising physical suggestion boxes and digital systems should be constructed, with a timely response commitment system. Efforts should focus on expanding students’ academic horizons and cultivating innovation capabilities, building industry-university-research collaborative platforms based on disciplinary strengths, and establishing specialised departmental research funds. Master’s students participating in international academic conferences, discipline competitions, and achievement transformation projects should receive graded financial support and joint training from supervisory teams, creating a comprehensive and sustained support chain.

Thirdly, master’s students should set clear academic research goals and dynamically optimise their skill matrices for academic growth, actively seeking institutional, supervisory, and peer support resources. By enhancing psychological resilience, cultivating a positive research attitude, relieving stress appropriately, and enhancing self-control abilities, students can adjust their research strategies timely. Additionally, building effective interpersonal networks, maintaining an optimistic mindset, and seeking professional psychological and academic support help students better handle research challenges, fostering academic growth and advancement. Individual self-development and adjustment are key to mitigating anxiety stemming from academic research pressure.

Fourthly, supervisors should provide master’s students with adequate psychological support alongside academic guidance. Clear research plans and goals should be formulated jointly with students, coupled with regular research progress reviews, timely feedback, and constructive guidance, thereby creating a harmonious research environment. Supervisors should foster positive research cultures within research groups, closely monitor students’ emotional states, communicate promptly when negative emotional reactions arise, and assist students in exploring coping strategies. Cultivating healthy research habits and guiding students towards positively confronting challenges can reduce anxiety resulting from uncertainty. Supervisors play a crucial role in alleviating master’s students’ academic anxiety.

This study, conducted under the context of involution, explored the mechanisms by which dual academic research pressure affects anxiety among master’s students in China, based on dual-pressure perception theory. It offers new empirical perspectives for examining the relationship between postgraduate academic research pressures and anxiety, providing practical insights for interventions targeting postgraduate anxiety. However, limitations are acknowledged. Due to the cross-sectional design adopted, causal relationships cannot be inferred solely from the current findings. Further exploration of causal associations between variables requires experimental and longitudinal research to deepen understanding of their interaction mechanisms. Although multiple data collections were performed, variable assessment was primarily conducted by the research team independently, potentially introducing common-method bias. Future research should continuously improve evaluations of innovative behaviours, recommending the exploration and application of more novel and rigorous assessment standards.

## 6 Limitations and future research

Within an involution context, this study empirically elucidated the mechanisms by which dual academic research pressures shape dual-dimensional anxiety among Chinese master’s students, offering important theoretical insights for localised academic-stress interventions. Future inquiries may be deepened as follows. Although a three-wave tracking design was adopted, which to some extent mitigated common-method bias and revealed temporal ordering, causal chains should still be inferred with caution. Future work could employ diary methods or experience sampling methods to capture the momentary dynamics of stress appraisal, resource depletion, and anxiety during daily research tasks. Experimental designs could also be introduced to manipulate challenge-type and hindrance-type tasks in laboratory settings. Secondly, the core variables were all self-reported by the same respondents. Future studies could incorporate more objective indicators to enrich validity—for example, combining physiological indices when measuring anxiety, and introducing cross-ratings by supervisors or peers when assessing achievement motivation. Finally, the theoretical construction of the involution context should be deepened. Although this study innovatively took involvement as a macro-level theoretical backdrop, it was not operationalised as a measurable variable within the model. This provides an opportunity for future cross-cultural research. Subsequent studies should endeavour to develop a localised involution-perception scale and introduce it as a higher-order contextual moderator.

## Data Availability

The original contributions presented in the study are included in the article/supplementary material, further inquiries can be directed to the corresponding author.
